# The “Who” System of the Human Brain: A System for Social Cognition About the Self and Others

**DOI:** 10.3389/fnhum.2020.00224

**Published:** 2020-06-19

**Authors:** Steven Brown

**Affiliations:** Department of Psychology, Neuroscience & Behaviour, McMaster University, Hamilton, ON, Canada

**Keywords:** self, other, character, narrative, brain, theory-of-mind, mentalizing, mimicry

## Abstract

Neuroscientists are fond of talking about brain systems for the processing of “what” and “where” information about objects and their locations. What is critically missing is the concept of a “who” system dedicated to the neural processing of information about social agents—both the self and others—and their interactions. I propose here the characterization of such a system, one that functions not only in perception but in production as well, such as when recounting stories about oneself and others. The most human-specific features of the “who” system are two complementary systems that I refer to as the *other-as-self* mechanism and the *self-as-other* mechanism. The major function of the other-as-self mechanism is to perceive other people egocentrically as proxies of the self, as occurs through the processes of mentalizing and empathizing in both everyday life and in the experience of the theatrical and literary arts. The major function of the self-as-other mechanism is to overtly depict other people during acts of communication through vocal and gestural processes of mimicry, such as occurs during quotation in conversation and through acting in the theatrical arts. Overall, the “who” system of the human brain mediates both perceptual and behavioral aspects of social cognition, and establishes the existential distinction between self and other in human cognition. I present neural models for the instantiation of the “who” system in the human brain and conclude with a discussion of how narrative serves as a foundation for human cognition more generally, what I refer to as narrative-based cognition.

## Introduction

Neuroscientists since the 1980s have been fond of talking about neural systems for the processing of “what” (object identity), “where” (object location), and “how” (sensory-guided motor activity) information in the brains of humans and non-human animals (Mishkin and Ungerleider, 1982; Mishkin et al., 1983; Goodale and Milner, 1992). What has been strongly lacking is a neural system for the processing of “who” information about conspecifics as social agents, despite a large impetus to develop a field of social neuroscience (Cacioppo et al., 2010). It should be possible to take advantage of findings from social neuroscience and consolidate them into a unified model of a “who” system in the brain, in other words a neurocognitive system for processing information about other people and the self as social agents. Such a system should be involved in: (1) distinguishing the self from others; (2) establishing the different personas of the self that occur in different social situations; (3) identifying other people based on both individual and group traits; and (4) classifying them as either friends or foes in the drama of social life. Friends are people who are liked, trusted, cared about, and sought out. Foes are people who are disliked, mistrusted, feared, and actively avoided. A critical function of a “who” system is to establish the *social* *status* of other people in relation to the self as characters in the drama of one’s life.

While the study of social cognition has put an overwhelming emphasis on perceptual processes, a more balanced approach to the topic must include the production component of social cognition as well. An analysis of production processes permits an exploration of not only social behavior, including all aspects of social interaction, but of the importance of role-playing in human life, a topic that has garnered little attention in either psychology or neuroscience (Brown, 2017), but which has acquired a strong historical presence in the study of acting theory since the 18th century (Diderot, 1783; Stanislavki, 1936, 1949; Kemp, 2012). An understanding of the core process that underlies acting, namely personal mimicry, provides an evolutionary unification of the two novel capacities for vocal imitation and gestural imitation that emerged during human evolution, even though the two are almost always discussed in separate literatures.

In developing the concept of a “who” system in the brain, I want to ground this system in the cognitive neuroscience of *narrative*, and argue that a narrative approach provides the best means for understanding “who” functioning in human cognition. I will capitalize on two major traditions in doing so. They reflect ideas related to character and plot, respectively, which are the two major components of narrative. First, the dramaturgical approach to social psychology (Goffman, 1959; Shulman, 2017) argues that the self and others can be conceptualized as a series of characters engaged in a web of social interactions driven by dramatic considerations. Individuals play different roles in different social situations based on the people they are interacting with at the time. Goffman (1959) focused his analyses on the role-playing that takes place in various professions and pointed out that such role-playing occurs in specific settings, that the players wear costumes specific for their role (e.g., the lab coat of a doctor), and that they employ props appropriate for the role (e.g., a stethoscope). In other words, social interaction is a form of stagecraft. From a dramaturgical standpoint, certain interaction partners are friends who support our goals by cooperating with us, while others are antagonists or competitors who obstruct us by providing obstacles to our goal achievement.

Second, Bruner (1986) argued that social cognition is mediated by a specialized mode of causal inference—what he called the “narrative mode”—that is distinct from the scientific mode that dominates our mechanistic understanding of non-social phenomena in the world. While the latter uses principles of physical causation to explain natural phenomena, the narrative mode of inference operates by conferring causal efficacy onto psychological states—for example, intentions, emotions, desires, goals, beliefs—in explaining the behavior of the self and other people, an idea that dominates folk psychology (Hutto, 2007). This is a distinctly social mode of cognition that is specifically dedicated to processing “who” information about social agents. The narrative mode can be thought of as the who system’s “why” mechanism (Spunt and Lieberman, 2014); it is its causal mechanism for explaining the behavior of others and the self with reference to psychological states. Developmentally, this emerges through children’s widespread engagement with fictional scenarios in their daily lives (Hutto, 2007). Children learn not only about physical causality through an exploration of fictional worlds (Walker et al., 2015; Hopkins and Weisberg, 2017), but about psychological causality as well, such as the kind that underlies social reasoning in stories with moral lessons (Hopkins and Weisberg, 2017; Walker and Lombrozo, 2017).

A means of unifying these two perspectives—the dramaturgical perspective that social behavior is a form of role-playing and the proposal that social cognition is predicated on a narrative mode of inference that confers causal efficacy onto psychological states—is by considering social cognition to be mediated by *plot-schemas*, in which the behavior of the self is quite similar to that of a protagonist in a story. Much of human behavior is goal-directed, and such behavior is often hindered by obstacles, not least social obstacles created by antagonistic individuals or institutions. These obstacles trigger problem-solving strategies that aim to overcome the antagonism to achieve one’s goal, oftentimes with the help of people who serve as supporters and enablers (Tu and Brown, in press). The result can be either a positive or negative outcome for the person, either terminating the plot or stimulating new attempts at solving the problem. Hence, goal-directed behavior often plays out in a plot-like manner in the context of a cast of characters, some of whom are antagonistic to and others of whom are supportive of the social agent. In the concluding section of the article, I will present a model of what I call narrative-based cognition (NBC) that proposes that goal-directed behavior can be thought of as being “a story in the making” from the standpoint of the social agent.

## The Standard Mode of Social Cognition

**Figure 1:**
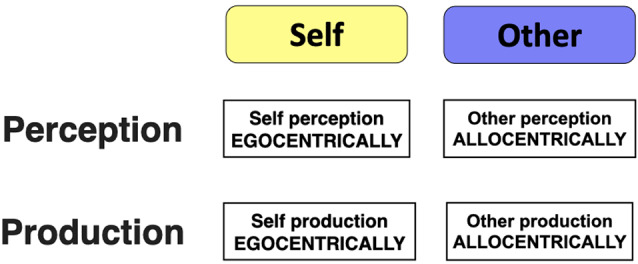
The “standard” mode of social cognition. The standard mode of social cognition is defined as that mode in which the self is processed egocentrically and the other is processed allocentrically, both in perception and production.

Throughout this article, I am going to consider a contrast between a “standard” mode of social cognition and what I will refer to as the “narrative” mode, in keeping with Bruner’s terminology. These modes will be described in detail in the following sections. Both of them are predicated on the central importance of perspective-taking in human cognition. This can be described in at least two different manners. Literary theorists distinguish the *first-person* (1P) perspective of the self from the *third-person* (3P) perspective of the other. By this analysis, theory-of-mind is the process of adopting a 3P perspective on someone. A second manner of describing perspective-taking comes from the study of spatial cognition. Here one finds a distinction between the *egocentric* perspective, where spatial processing occurs from the internal corporeal perspective of the self (as in looking out through one’s eyes), and the *allocentric* perspective, where spatial processing occurs with respect to some external frame of reference, most commonly a Cartesian coordinate system (Klatzky, 1998; Mellinger and Vosgerau, 2010; Gramann, 2013). Combining these two approaches to perspective-taking, we can see that the 1P perspective is generally egocentric, while the 3P perspective is generally allocentric. Relations of this type define what I will call the standard mode of social cognition. Figure 1 provides a conceptual description of the standard mode, covering both perception and production. Its 2 × 2 structure leads to four psychological processes:

### Self-perception

This includes the fundamental process of self-awareness that forms the basis of consciousness and self-identity. Such self-awareness includes not only knowledge of one’s fluctuating psychophysiological states—including emotions, feelings, desires, intentions, goals, and so on—but also knowledge of one’s enduring trait features, including physical and personality features.

### Self-production

The dramaturgical paradigm that was just discussed (Goffman, 1959; Shulman, 2017) posits that people present themselves during social interactions as a series of *personas* that vary in behavioral features as a function of social context and interaction partners. Such personas are not true characters (i.e., other people), but are instead variants of a multifaceted self. For example, a given individual can play the roles of mother, daughter, boss, customer, patient, and wife in different contexts as a function of the social situation and the interaction partners. Each of these personas is associated with a particular set of behaviors and social traits that reflect, in part, the social hierarchy of the culture and the inherent status and power relationships of that hierarchy (Brown, 2019). These include interactive roles related to coordinative behaviors, as seen in the group-wide dancing that occurs in ceremonial rituals in indigenous cultures, where status relationships are reflected in the coordinative roles of leader, follower, and coequal.

### Other Perception

“Other” processing in the standard mode of social cognition involves allocentric processing of others in both perception and production (see the right side of Figure 1)^1^. At the most fundamental level, this involves the perception of people’s trait features from an allocentric perspective. A critical part of “who” functioning in cognition is the ability to recognize *who someone is*, that is to identify both the person’s group memberships (e.g., gender, age group, family relationships) and their identity as an individual. I will consider four types of features that contribute to the social identification of individuals, and they apply as much to the self as to others. First, there are trait features that are perceived statically because they are either invariant or that they change very slowly over time. These include physical features such as gender, ethnic features, age group, height, facial structure, body size, and strength, among many others. Neuroscientists have proposed that there are specialized brain modules for the perception of faces, voices, and bodies, specializations that are devoted to social cognition (Schirmer and Adolphs, 2017). Second, some features are perceived dynamically, such as facial expressions, vocal prosody, and body movements and actions, that we can use to recognize who somebody is. Third, beyond physical and behavioral features alone, the perception of other people focuses on their psychological and social traits, including enduring features such as personality traits or more-variable features like social status. Within social psychology, the so-called Big 5 personality dimensions of conscientiousness, agreeableness, neuroticism, openness, and extroversion have been widely discussed as enduring trait features of people (Digman, 1990). Berry and Brown (2017) demonstrated that literary characters could be successfully classified in a two-dimensional manner according to the orthogonal personality dimensions of assertiveness and cooperativeness. Also, they formulated the concept of an “ethotype” (where the root “etho” means character) to connote a personality variant of a literary character, analogous to the way that persona describes variants of the self. For example, the character of the king can be virtuous or authoritarian. Likewise, the cynic can be a persnickety ogre or an elegant dandy, and such ethotypes should undoubtedly vary in their physical traits as well.

Fourth, the perception of other people in the standard mode of social cognition engages systems of emotional appraisal, especially as it pertains to interpersonal status relationships. These appraisals vary as a function of valence with regard to whether we see others as people whom we like, trust, and want to cooperate with (positive valence) or people whom we dislike, fear, and attempt to avoid as threats (negative valence). Dramaturgically, this creates a divide between supporters and antagonists, respectively, helping to establish interactive roles between the self and others in social dramas. This can be seen quite clearly in connection with status relationships with others. Some people are perceived as more dominant than ourselves (e.g., parents, group leaders), others as more submissive (e.g., children, the infirmed), and yet others as equals (e.g., friends, colleagues). Two of the most important social appraisals that we make of other people are moral appraisals of the propriety of their actions and aesthetic appraisals of their attractiveness (Ortony et al., 1988). Moral emotions play a central role in dramaturgy since we tend to make positive moral appraisals of supporters and friends, and negative moral appraisals of antagonists whom we perceive as threatening. Hence, the status distinction between friend and foe is a critical feature of what a “who” system is designed to achieve in establishing social relationships and status hierarchies. Overall, allocentric perception of others focuses on a combination of trait perception (physical, psychological, social), action perception, and emotional appraisals.

### Other Production

By “production,” I am referring here to communicative processes that occur during social interactions, not just to involuntary emotional expressions. The production side of “other” processing in the standard mode of social cognition is the production analog of trait perception, which I will refer to as *description*. At the most basic level, this involves a description of people’s trait features or actions, as conveyed through language, gesturing, or graphic-image generation. Later in the article, I will contrast description with *narration*, where narration incorporates a mentalistic understanding of people’s intentionality and agency, as based on the narrative mode of inference using theory-of-mind mechanisms. Description in the standard mode of social cognition is a more constrained process, one that is limited to *non*-mentalistic production processes, as related mainly to a description of people’s trait features, be they physical, psychological, or social. Description can also include a basic understanding of actions and events, but it should preclude an analysis based on people’s intentionality, emotions, and other psychological explanatory factors that are the purview of the narrative mode of cognition.

## The Narrative Mode of Social Cognition: The Other-as-Self and Self-as-Other Mechanisms

I have defined the standard mode of social cognition as that psychological mode in which self-related processing occurs egocentrically and other-related processing occurs allocentrically (Figure 1). The current section examines mechanisms by which social cognition can move psychologically beyond the confines of these perspectives and help establish several human-specific features of social cognition. These mechanisms will comprise the narrative mode of social cognition. If the standard mode emphasizes the mechanisms that underlie our ability to socially identify other people—in other words, to recognize *who* someone is—then what I am calling the narrative mode moves beyond this recognition process to allow people to both get into the mind of another person through mentalizing and to portray another person through mimicry. I will talk about the two principal mechanisms of the narrative mode as being the “other-as-self” (OS) mechanism and the “self-as-other” (SO) mechanism. They reflect the two major modalities of storytelling outlined by Plato (1968; 380BCE) in *The Republic*, namely diegesis and mimesis. In diegesis, the characters of a story are described by a narrator, whereas in mimesis, they are conveyed through impersonation by actors. The OS mechanism is diegetic, while the SO mechanism is mimetic.

### The Other-as-Self (OS) Mechanism

**Figure 2:**
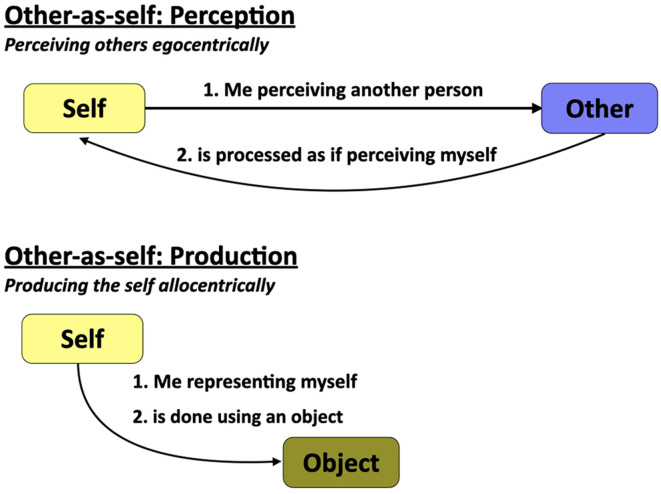
The other-as-self mechanism. The principal other-as-self mechanism is found in perception (top panel): perceiving others egocentrically through mentalizing processes. A second, though uncommon, mechanism is found in production (bottom panel): producing the self allocentrically. This can occur as a projection of the self onto a self-avatar or other object, for example in virtual reality applications. The term “object” is used here to refer to either a physical or virtual representation of the self.

We can perceive other people, not just in standard allocentric terms with reference to their observable physical traits and actions, but also *egocentrically* as people like ourselves whose unobservable mental states we can infer, understand, and share in through theory-of-mind and empathy mechanisms. OS functioning is the ability to perceive other people as proxies of oneself. The top panel of Figure 2 outlines the logic of the predominant form of OS processing, namely the ability to perceive others egocentrically through mentalizing. It states that “me perceiving another person is processed as if perceiving myself.” I will argue in the neuroscience section below that this process is mediated by a mirror-type neural mechanism that underlies ostensive communication in humans. *Mentalizing is the most basic other-as-self function in human cognition*. Mentalizing—also known as theory-of-mind and mindreading—is a process by which a person mentally looks beyond him/herself to develop inferences about the thoughts, beliefs, desires, intentions, and emotions of another person (Frith and Frith, 2003; Nichols and Stich, 2003). It is the ability to see other people in egocentric terms as proxies of the self and to understand their psychophysiological states through cognitive mechanisms of mental stimulation, or what some have referred to as “mental mind travel” (Ferretti et al., 2017). It is a process that is deficient in individuals with autism, who have a reduced ability to mentally extend beyond themselves and to think about people’s mental states, including their own (Williams, 2010).

Mentalizing is the instantiation of the narrative mode of causal inference in social cognition, where social phenomena are understood and explained with reference not to physical causation but instead to mentalistic causation related to people’s intentions, desires, beliefs, and emotions. As mentioned earlier, the narrative mode comprises the who system’s “why” mechanism; it allows people to explain the behavior of others using principles of psychological causation, rather than physical causation. Importantly, mentalizing is not merely about making an inference about another person’s mental states, but about translating these mental states into egocentric terms as states that we ourselves can understand and engage in. This is relevant not only for the perception of people during standard social interactions but also for the perception of characters in the theatrical and literary arts, with whom people readily engage psychologically (Fludernik, 1996; Oatley, 1999; Palmer, 2004; Zunshine, 2006; Mar and Oatley, 2008; Hogan, 2011; Herman, 2013). Hence, this is a major mechanism for narrative perception and perspective-taking in the arts.

The extreme version of OS perception is empathy, in which a person not only *infers* information about another person’s emotions but *feels* the emotions assumed to be felt by that person (de Waal, 2008; Hatfield et al., 2009). Empathy is the contagious process of sharing in someone’s psychophysiological experience at the same time that they presumed to. In empathy, we perceive others egocentrically to the point that we vicariously experience their emotions and bodily states (Paulus et al., 2013). Empathy can be conceptualized in OS terms as a process of engulfment. It is as if my skin has somehow wrapped around your skin such that, if I see someone placing a pin into your skin, I feel your pain at the same time that you are presumed to be feeling it. Through this process, you become an extension of my own body, a part of me.^2^ The result is a state of shared emotional experience. There are several distinctions in the psychology literature between mentalizing as a process of mere awareness of someone’s emotions and empathy as a process of actually feeling someone’s emotions. These include sympathy vs. empathy, cognitive empathy vs. affective empathy, cognitive empathy vs. compassionate empathy, and cold empathy vs. hot empathy (Davis et al., 1987; Shamay-Tsoory, 2011; Winner, 2019). To my mind, terms like cognitive empathy and cold empathy are inaccurate descriptions of the concept, since mentalizing without empathy does not qualify as empathy, which is a process of contagiously sharing in the emotions of another person, not just developing a cognitive awareness of or intuition about that person’s emotions. What this implies is that most psychological models of empathy operate hierarchically such that the cognitive (mentalistic) process of inferring an emotional state is seen as being a prerequisite to the affective (physiological) process of feeling an emotion.

While the OS mechanism just discussed is perceptual, its discursive counterpart creates a significant innovation in the capacity for communication in human cognition, namely *narration*. Narration permits a transition from a non-mentalistic process of description based on people’s observable trait features and actions to a mentalistic and agentic process in which the behavior of other people is described and explained with regards to their intentions, desires, beliefs, and emotions. In other words, narration is a description of others and the self using the mentalizing-based narrative mode of inference, rather than the scientific mode based on descriptions of observable traits and actions alone. It is a process of “narrativizing” human behavior in terms of mentalistic and intentional causes, about conveying the “why,” not just the “how,” of human behavior. Importantly, *narration is the production analog of theory-of-mind*. If theory-of-mind is the covert process of inferring a person’s desires, motivations, intentions, beliefs, and emotions, then narration is the public process of externalizing and depicting such psychophysiological states during acts of social communication (Clark, 2016). The neuroscience section below will discuss the fact that narration (as production) and mentalizing (as perception) take advantage of a shared sensorimotor mentalizing system that is comparably active in production and perception, suggesting a model in which theory-of-mind and narration co-evolved during human evolution, doing so *via* a mirror-system mechanism.

Narration permits the emergence of stories as a communicative phenomenon in human evolution (Gottschall and Wilson, 2005; Boyd, 2009, 2018), a significant innovation in social cognition and a major force in cultural evolution. Through narration, we can communicate psychologically about other people and the self as social agents driven by desires and intentions. In its richest forms, narration is not only able to convey information about people as they currently are, but also about their past behaviors and potential future actions. As Aristotle pointed out in *Poetics*, “… the function of the poet is not to say what has happened, but to say the kind of thing that would happen, i.e., what is possible in accordance with probability or necessity” Aristotle (1996; 16; 335BCE). In terms of real life, this amounts to a process of prediction and simulation about the probable behaviors that people will carry out based on psychological inferences about their desires, intentionality, and emotional states. This underlies the unlimited potential of writers of literature and drama to creatively invent story-worlds, characters, and plot scenarios that are fictional yet still realistic (Hogan, 2013).

A critical part of narration is a process that I call “protagonism” (Brown et al., 2019a), also referred to as focalization in literary theory (Abbott, 2008). Protagonism is the fundamental narrative function of establishing a single person’s perspective in a story, namely that of the protagonist. In real life, events typically involve multiple individuals or even masses of individuals. While histories and news stories have mass protagonists, personal and literary stories do not. Personal stories are always protagonist-centered. They describe events from the standpoint of one person’s interests and welfare. Protagonism is about carving out that person’s perspective and distinguishing it from the perspective of all of the other agents taking part in the event. For self-related stories (1P perspective), we ourselves are the protagonist (Sarbin, 1990; Labov, 2001; Bauer et al., 2008; De Fina and Georgakopoulou, 2012). For stories about other people (3P perspective), we select one particular other for this role and describe outcomes in terms of his/her interests and welfare. However, the critical caveat here is that the other-as-self mechanism ensures that narration operates by developing insights into other people as relatable self-proxies (Storm, 2016). In other words, the protagonists of stories are people like ourselves who we can relate to egocentrically. Hence, whether in production or perception, protagonism defines the protagonist’s perspective for a narrative. Producers and perceivers of stories latch onto the perspective of that one character and exclude all others. In the vast majority of stories, this is presented as an antagonism between a protagonist and other individuals or institutions having competing interests with him/her (Abbott, 2008). This is a critical facet of how we view social phenomena as plot-schemas and another manner in which personal stories are different from news stories.

The lower part of Figure 2 shows a second, though relatively uncommon, the process of OS functioning, one that occurs in production, rather than perception. The logic of this process is that “me representing myself is done with an object,” rather than egocentrically with my own body. A contemporary example of this is the use of avatars of the self in virtual reality chat rooms and role-playing video games (Caracciolo, 2015). In such situations, a person has to project him- or herself onto an avatar, resulting in an other-as-self process in production. A more common example is found in pantomime. Consider the pantomime of someone walking in which the mimer represents herself walking down the street not by performing a full-body egocentric gesture the way that a mime actor would, but instead by using the index and middle fingers of her hand to represent her two legs walking along the street. This is referred to in the gesture literature as a body-part-as-object (BPO) pantomime (Boyatzis and Watson, 1993; Suddendorf et al., 1999) since the mimer’s fingers undergo a process of object substitution to become legs. In such a pantomime, the self is represented in production in an allocentric manner, which is a deviation from the self → egocentric processing of the standard mode of social cognition.

### The Self-as-Other (SO) Mechanism

**Figure 3:**
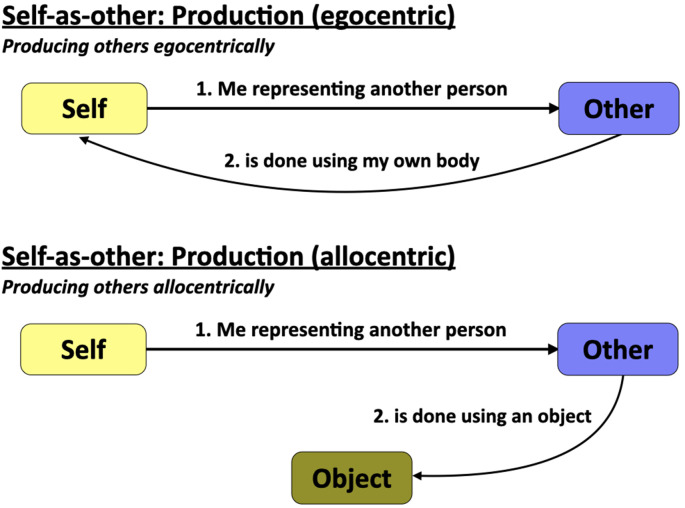
The self-as-other mechanism. The principal self-as-other mechanism involves portraying others egocentrically through mimicry and acting (top panel). A second, though uncommon, mechanism involves producing others allocentrically (bottom panel). This can occur as a portrayal of another person using an object, for example a dummy in ventriloquism or an avatar in role-playing video games. The term “object” is used here to refer to either a physical or virtual representation of another person.

If the predominant form of OS functioning is the perception of others as proxies of the self through mentalizing, then the predominant form of SO functioning is the portrayal of others using the self as a vessel, in other words *producing others egocentrically* through mimicry and acting. Note that I am using the term mimicry in a broad sense throughout this article to refer to the impersonation both of familiar individuals and dramatic characters. Mimicry is being used in the Platonic sense of likening oneself to another person, rather than of producing a replica of some known person or entity. In SO functioning, the self is transformed, either partially or wholly, into another person. The top part of Figure 3 outlines that the logic of the SO mechanism is that “me representing another person is done using my own body.” This occurs *via* gestural and vocal mechanisms of personal mimicry, such as that which occurs during quotation in conversation (Bavelas et al., 2014; Blackwell et al., 2015; Stec et al., 2016), during the impersonation of characters in bedtime-story reading (Matharu et al., in press), and in the performative context of the theatrical arts (Konijn, 2000; Kemp, 2012; Schechner, 2013). While dramaturgical theory in social psychology tells us that we present different personas of our self in different social situations, there are also occasions in which we portray *other people* during social interactions. In the extreme case, this is done by professional actors when performing the role of fictional characters in dramatic works as part of theatre performances. However, there is a large diversity of functions in which character portrayal occurs during everyday social interactions, what I have referred to elsewhere as proto-acting (Brown, 2017). These are processes of personal mimicry and character impersonation that occur in a far more transient manner than that which takes place during full-fledged theatrical performances. The most common form occurs when quoting someone in conversation, such as when a man raises the pitch of his voice when quoting his wife having said to him “Honey, did you take out the trash?”

I propose that, through the SO mechanism, another person comes to impinge on our self and take us over, leading to a retraction of self resources. Like all human beings, I have only one voice, one face, and one body. The more that I perform the gestures of another person through mimicry, the less that there is of the features of my voice, face, and body. Because of this resource competition, mimicry is a zero-sum game. While I can certainly alternate between myself and another person—much the way that a ventriloquist does with a dummy—I cannot be both of us at the same time. Resource limitations demand that mimicry be, at least to a significant extent, a retraction of the self. True acting is an extreme form of this, in which a character takes over a significant part of the actor’s self. By this line of reasoning, acting is akin to a process of possession, a phenomenon that is seen across world cultures in religious rituals in which participants come to be taken over by spirits, ancestors, and deities (Rouget, 1985; Schechner, 2013). Perhaps the main difference between spirit possession and acting is that, while actors maintain a stable sense of “split consciousness” between themselves and their character (Konijn, 2000; Kemp, 2012), participants in possession rituals can transiently cross the divide to the point that they have lost themselves and have become fully taken over by the spirits (Rouget, 1985), much as occurs in a more chronic and debilitating manner in psychiatric conditions of personality disorders. In such cases, identification with a character is complete, and there is a minimal splitting of consciousness between the self and the other. I will argue in the neuroscience section below that acting is, at least in part, a “loss of self,” and that this comes about through suppression of the intrinsic self-persona (Brown et al., 2019b). This process can become accentuated when performers employ acting methods that encourage deep self-identification with characters.

As mentioned in the “Introduction” section, character portrayal through personal mimicry creates a union between the two newly-evolved capacities for gestural imitation and vocal imitation found in humans. This is significant since gestural imitation is discussed in relation to language evolution separate from speech (Arbib, 2012), whereas vocal imitation is discussed in relation to speech evolution separate from language (MacNeilage, 2008). Therefore, the self-as-other mechanism of personal mimicry and proto-acting provides *an evolutionary unification of gestural and vocal imitative processes* for the first time (although see Donald, 1991 for related ideas). Proto-acting also establishes role-playing as a pervasive process in human social cognition. A universal ontogenetic manifestation of this is seen in the pretend play of children, where the participants portray characters in improvised dramas, often involving props (Walton, 1999; Harris, 2000). Some of these props also serve as characters in the drama, such as dolls and toy animals, which are brought to life through a process of animism. Compared to narration’s ability to depict other people from a third-person perspective, the self-as-other mechanism permits a depiction of people from a first-person perspective through the embodied process of impersonation, something that establishes the “fictional first-person” perspective of the actor (Brown et al., 2019b). Despite decades of work on theory-of-mind and its neural basis, there has been scant work on personal mimicry and character portrayal in the fields of psychology and neuroscience (Goldstein and Bloom, 2011), least of all in the field that calls itself social neuroscience.

The bottom panel of Figure 3 shows an additional, though uncommon, mechanism of SO functioning: producing others allocentrically using objects. The various SO functions discussed thus far—mimicry, acting, and possession—are all forms of character portrayal that use the full body of the self to impersonate the other in an egocentric manner. However, there are also allocentric means of impersonating the other, and that is with the use and control of objects. This can involve physical objects like puppets or dummies (Orenstein, 2017), or virtual objects like the computer avatars that are found in video games (Caracciolo, 2015). Compared to the egocentric forms of SO functioning, the allocentric forms create a triadic relationship between self, character, and object. Also, while virtually all forms of mimesis in the Platonic sense are performed egocentrically, character portrayal using an object as an implement is the only format of mimesis that is allocentric.

**Figure 4:**
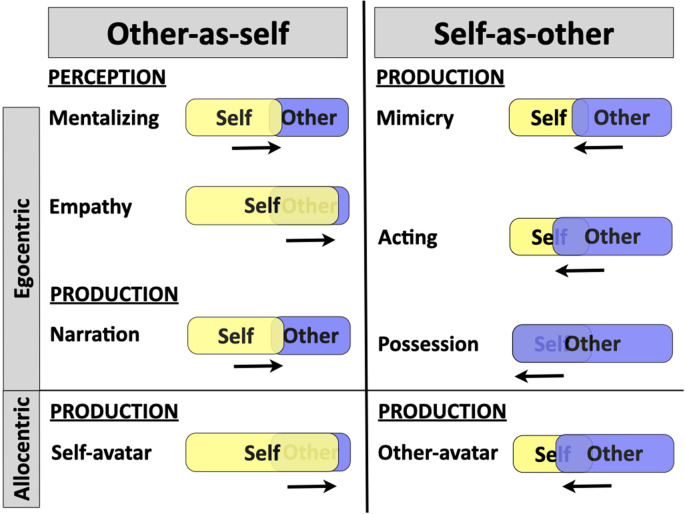
Summary of the narrative mode of social cognition. The principal other-as-self mechanism involves perceiving others egocentrically through mentalizing and empathy. A production counterpart occurs in the form of narration. A second mechanism works in production to represent the self allocentrically using objects such as avatars. The principal self-as-other mechanism involves portraying the other egocentrically through mimicry, acting, and occasionally spirit possession. A second mechanism works to portray others allocentrically using objects, such as puppets and avatars. The figure uses shading and arrows to provide an approximate representation of the dynamics of self and other processing for each mechanism. In mentalizing, empathy, and narration, the self reaches out psychologically to an other’s mind. In the use of self-avatars, the self gets projected onto an object. In mimicry, acting, and possession, an other comes to inhabit the self’s body during a performance. In the use of other-avatars, the portrayal of an “other” occurs using an object, rather than the self’s body. Note that the processes shown horizontally on either side of the central vertical divider are not meant to be paired with one another; the organization is arbitrary.

Figure 4 provides a combined summary of the OS and SO mechanisms, both egocentric and allocentric forms. The figure proposes a rough directionality of the effects between self and others using shading and arrows. The major form of OS functioning is mentalizing, in which the self reaches out to perceive the mind of the other. Narration is the production analog of this. Empathy is a more extreme form of this, in which the self engulfs the other and shares in that person’s psychophysiological states. The allocentric form of OS function is the projection of the self onto self-avatars, as in virtual reality chat rooms. For SO functioning, the egocentric forms are shown as progressive retractions of the self by the other, from simple mimicry to dramatic acting to spirit possession. The allocentric form of SO is the use of other-avatars, either physical or virtual, as representations of others, as seen in ventriloquism, puppet theatre, and role-playing video games. A basic proposal of the current model of human cognition is that the evolution of the sense of self comes about not just by the ability to perceive the self egocentrically (i.e., self-awareness), but by the ability *to perceive and produce others egocentrically through mentalizing and mimicry*, *respectively*.

Before concluding this section, I would like to apply the reasoning developed here to the cognitive basis of religion, in particular to the observation that *gods are typically seen as characters in the drama of human life* and are thus subject to the operations of the “who” system. Gods are frequently conceived of as personified beings, as in the case of the Judeo-Christian God, who is seen as a male parental figure. In polytheistic religions, some gods function as supporters and protectors, while others function as antagonists, just as in drama. Even in folklore, there are supernatural beings that are the sources of good (e.g., angels, fairies) and those that are the sources of evil (e.g., demons, evil spirits). In many cultures, but by no means all of them, gods take the form of dead ancestors and hence have many of the salient physical and psychological attributes of human beings (Steadman and Palmer, 2008), including the ability to communicate with living humans using natural language. Gods are often portrayed as moralizing beings and authority figures who make emotional judgments about the actions of humans (Johnson, 2005, 2015). These are typically prosocial judgments that resemble the social appraisals that humans make of one another. Prayer provides an accessible means of communicating with gods using natural language. This can be done to make requests for desired things, to express thanks for requests that were granted, or to seek forgiveness for behaviors that might normally lead to punishment. In most cases, prayer is a monologic activity directed toward the god (even when it is done as group worship), although some people report hearing the voice of a god talking back to them or seeing signs that the god has responded to a request that occurred in a prayer. This strongly suggests that prayer is a process that has a clear mentalizing component to it, as if people are trying to read the mind of a god while talking to it and are engaging in processes of persuasion. It is not surprising, therefore, that conceptions of gods often have a strongly egocentric form to them (Epley et al., 2009), reflecting the person’s own conception of themselves (i.e., other-as-self).

## The “Who” System of the Human Brain

Just as neuroscientists talk about “what,” “where” and “how” systems in the brain, I would like to suggest that the brain also contains a “who” system for social cognition and that this system operates by processing information about others and the self as actors in social dramas. The “who” system should mediate both the standard mode and the narrative mode of social cognition. The core of the “who” system is comprised of a well-described neural system that processes mentalistic information about both oneself and other people. It is generally referred to as *the mentalizing system* (Frith and Frith, 2003, 2006; Spunt and Lieberman, 2013), which is important both for self-awareness and for creating inferences about the mental states of others, including their beliefs, intentions, desires, and emotions. This system functions as a hub for many aspects of social cognition. The mentalizing system strongly overlaps with the “default mode network” involved in developing a sense of internal awareness about the self, as opposed to an external awareness of objects in the environment (Andrews-Hanna et al., 2010; Mars et al., 2012), where the latter is much more the domain of the “what,” “where,” and “how” systems.

**Figure 5:**
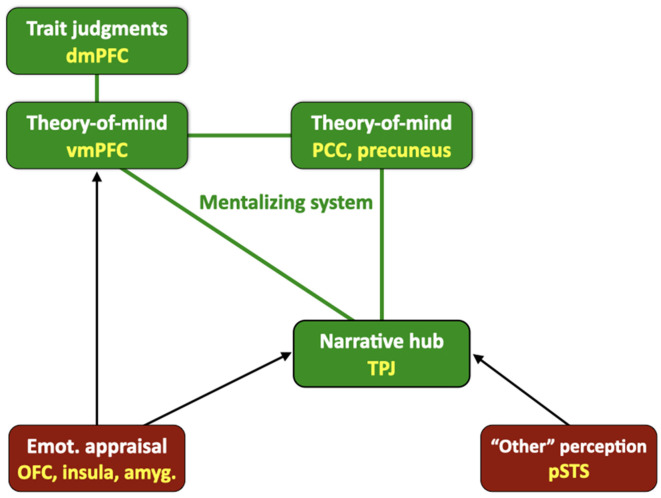
A neural model of the “who” system of the human brain. While “who” processing involves all facets of sensory processing about physical features and movement across the five major senses, the core of the who system is a network for character processing. It maps onto systems for mentalizing, trait processing, emotion perception, and emotional appraisal. The mentalizing components include the ventromedial prefrontal cortex (vmPFC), posterior cingulate cortex (PCC), precuneus, and the cortex of the temporoparietal junction (TPJ), which acts as a hub that interfaces areas for processing emotional appraisals [orbitofrontal cortex (OFC), insula, and amygdala (amyg.)] and the perception of emotional expression (the posterior superior temporal sulcus, pSTS), as well as episode processing for plot-schemas that is not shown in the figure. Trait processing includes areas in the dorsomedial prefrontal cortex (dmPFC). Components of the mentalizing system are shown in green, while sensory and emotion areas are shown in red. The figure is oriented anatomically such that the left side of the figure corresponds with the anterior part of the brain. Abbreviation: Emot., emotional.

Figure 5 shows schematically that the core of the “who” system is made up principally of medial cortical structures, including the ventromedial prefrontal cortex (vmPFC) and posterior cingulate cortex (PCC), but also the precuneus, and potentially the retrosplenial cortex. The only major component of the mentalizing system that is on the lateral surface is the temporoparietal junction (TPJ), although several models also include the anterior temporopolar cortex (ATPC) as well. Another medial brain area that I include in this system is the dorsomedial prefrontal cortex (dmPFC), which is less involved in acquiring mentalistic knowledge as in understanding the trait features of oneself and others (Benoit et al., 2010; Ma et al., 2012; Garrison et al., 2015), including physical characteristics, which is another critical facet of who processing. The overlap between the mentalizing network and the default mode network occurs more so in the posterior regions of the TPJ and PCC than in the anterior region of the vmPFC (Mars et al., 2012).

Two kinds of areas interact with this mentalizing core: first many sensory areas that contribute to “allocentric perception of others” (Figure 1), as is key for the operation of the standard mode of social cognition, and secondly emotion areas that process moral appraisals regarding the social status of others in relation to the self, i.e., whether someone is a friend (resource) or a foe (threat) in the narrative sense, including ingroup vs. outgroup status. Regarding the first type of area, information about the trait features, actions, and expressive behaviors of other people is processed in higher-order sensory areas in the posterior part of the brain, including modules devoted to the perception of facial structure (fusiform face area), facial expressions (posterior superior temporal sulcus, pSTS), voices (voice-selective temporal-lobe areas), vocal prosody (pSTS), body structure (fusiform body area, extrastriate body area), and expressive movement patterns (area V5 and the pSTS; reviewed in de Gelder et al., 2015; Schirmer and Adolphs, 2017). The pSTS, which is shown in the figure, serves as a multisensory convergence point for perceiving vocal, facial, and bodily forms of emotional expression.

The second group of brain areas that interact with the mentalistic core of the “who” system is comprised of limbic emotion areas that mediate appraisals about the social status of others with regards to the self, not least whether someone is a friend or a foe. These are systems that work to help organisms recognize threats in the environment, but in this case they apply to *social* threats within and between social groups. For people whom we appraise as friends, we are inclined to cooperate with them, help them, share with them, trust them, sympathize and empathize with them, and engage in approach behaviors toward them. For people whom we see as foes, we have the opposite emotional profile and tend to engage in avoidance behaviors in relation to them. Interactions with friends are rewarding, while interactions with foes are threatening. Brain areas of relevance to status appraisals include the amygdala and insula for negative (threatening) social appraisals, and the TPJ and ventral striatum for positive social appraisals. Both sets of areas interact with the orbitofrontal cortex and vmPFC, which are involved in creating social appraisals and valuations more generally. Looking at the network in Figure 5 as a whole, the TPJ (and adjacent angular gyrus) is a good candidate for being a *narrative hub*, since it is involved in numerous functions that support narrative cognition, including language semantics (being a component of Wernicke’s area), speech processing (most especially phonological processing *via* the dorsal speech pathway), mentalizing (being a component of the mentalizing system), and social cooperation (Carter and Huettel, 2013; Strang and Park, 2017; Patel et al., 2019). In the remainder of this section, I will discuss key properties of the “who” system of the human brain that are relevant to its general function as a narrative system about the self and others as characters in social dramas.

### The Overlap Between Production and Perception

A handful of studies have directly compared the production and perception of narratives in the same participants. Both Silbert et al. (2014) and AbdulSabur et al. (2014) directly compared narrative production with narrative perception and found an overlap between the two in a large number of areas. Among them are all of the areas shown in Figure 5, including the TPJ, pSTS, mPFC, and PCC. As mentioned in the section about OS functioning above, I argue that narration is the production analog of theory-of-mind as a perceptual capacity. If theory-of-mind is the covert process of inferring a person’s motivations, beliefs, and emotions, then narration is the public process of externalizing such motivations, beliefs, and emotions through depictive acts of communication (Clark, 2016). Narration takes advantage of the other-as-self system for mentalizing, making narration an intrinsically mentalistic and thus character-centered process. This model implies that *theory-of-mind and narration co-evolved* as a coupled sensorimotor mechanism during human evolution. It is interesting to point out that, from a narrative standpoint, the system that mediates mentalizing is also involved in episodic memory about past personal episodes and prospective thinking about future personal actions (Buckner and Carroll, 2007; Spreng and Grady, 2010), suggesting that the mentalizing system truly is a narrative system.

An important implication of this overlap between the production and perception of narrative processing—as well as the proposal that social cognition operates according to a narrative mode—is that *the mentalizing system is the quintessential mirroring system in the human brain*. The standard view of a mirror system in the brain is based on the idea that observing a person’s action, especially a manual object-directed action, triggers a motor representation of that action in the perceiver’s brain (Rizzolatti and Craighero, 2004; Arbib, 2012). In other words, the perceiver develops an egocentric perspective on the actions of another person through motor simulation (Gallese, 2009). While this process has been discussed in great detail in the literature, I believe that the more significant effect of mirroring for social cognition is the achievement of an egocentric perspective on people’s *unobservable* *mental states—*not just their observable actions—and that this is far more the domain of the mentalizing system than the action-based mirror system. Vogeley (2017) proposed a useful functional comparison between the mirror neuron and mentalizing systems in which the mirror neuron system is involved in the social *detection* of spatial and bodily signals, while the mentalizing system is involved in social *evaluation* of the emotional and psychological states of others. This detection/evaluation dichotomy maps more or less onto my distinction between the standard and narrative modes of social cognition. Likewise, Spunt and Lieberman (2014) have described the distinction between the mirror system and mentalizing system in social cognition as that between “how” (behavior identification) and “why” (social causal inference), respectively.

The mirroring of intentional states is a critical prerequisite for the achievement of parity in the evolution of communication systems since such an evolution requires that all parties have a shared understanding of the communication signals and their referents (Arbib, 2012). Parity of this kind is based on *intersubjectivity*, which can be defined as “information processing allowing the exchange of inner experience in communicative acts” (Vogeley, 2017:2). The sharing of intentional states that the mentalizing system permits is far more central to the evolution of communication than are the functions of the hand-based mirror system, which has no particular connection with intentionality (although see Gallese, 2009, for an opposing view). A major function of the “who” system of the brain is to mediate the narrative mode of causal inference, in which behavior is understood by perceivers in mentalistic and intentionalistic terms, rather than in purely physicalistic terms. The narrative mode sets up the conditions for understanding the mental states of others and thus for achieving intersubjectivity between people. As a result, the mentalizing system, far more than the hand-related mirror system, is the mirroring system of greatest relevance for the evolution of human communication (Vogeley, 2017). The narrative mode of cognition is based not on motor simulation but on mental-state simulation. The metaphor of the mirror has far more significance when it comes to egocentric representations of intentions than to egocentric representations of visible hand actions.

### The Overlap Across Modalities of Production

The mentalizing system, as a sensorimotor system for “who” processing, interacts with multiple systems for overt expression, including the voice and the manual system for gesturing and tool use. This was explored across modalities of production by Yuan et al. (2018). In this functional MRI study, participants had to produce simple narrations using the “narrative triad” of speech, pantomime, and drawing. Narrations were based on headline prompts describing simple transitive actions carried out by a protagonist (e.g., “Surgeon finds scissors inside of patient”). As a control condition to eliminate modality-specific sensorimotor activations and to reveal the narrative areas of the network, participants had to describe the spatial properties of familiar objects (e.g., binoculars), without any reference to humans or human use. When the narration vs. description contrast was analyzed, the same set of mentalizing areas was observed for each of the three modalities of production, most notably the TPJ and PCC. This suggests that a common set of mentalizing areas mediates protagonist processing across the three production modalities of speech, pantomime, and drawing. Hence, not only is the mentalizing system activated in both production and perception (as described above), but it is jointly activated across various motor modalities of narration. This suggests that “who” processing is potentially amodal, interacting with modality-specific systems for the externalization and perception of narrative using a given sensorimotor pathway. Hence, the pleiotropy of the mentalizing system is demonstrated not only by production/perception overlap but by a cross-modal overlap in production as well.

### The Overlap Between Self and Other

The mentalizing system has historically been described as a system for 3P processing, in other words for inferring the mental states of other people through theory-of-mind mechanisms. However, the process of self-awareness is most likely mechanistically identical to inferring the mental states of others (Nichols and Stich, 2003). Consistent with this idea, almost all of the components of the mentalizing system are activated during *self*-processing tasks that involve mentalizing (Lombardo et al., 2010; Denny et al., 2012), suggesting that the network is more oriented towards the operation of mentalizing than to “other” processing *per se*. While a vast literature of studies has confirmed the importance of this network for mentalizing tasks, what has been far less clear is the *directionality* of the effects between 3P and 1P processing, except for the TPJ, which generally shows increased activity for 3P compared to 1P in direct comparisons (Ruby and Decety, 2003, 2004; Elliott et al., 2006; Lombardo et al., 2010; Rabin et al., 2010; Spreng and Grady, 2010; although see Vogeley et al., 2001; Schulte-Rüther et al., 2007). For midline structures like the mPFC and PCC, some studies show relative increases in the activation for 3P tasks compared to 1P tasks (e.g., Ruby and Decety, 2003, 2004; Seger et al., 2004; Elliott et al., 2006; Pfeifer et al., 2007; Lombardo et al., 2010), whereas other studies show relative increases for 1P compared to 3P (e.g., Kelley et al., 2002; Vogeley et al., 2004; Heatherton et al., 2006; Schulte-Rüther et al., 2007; Ames et al., 2008; D’Argembeau et al., 2008; Lombardo et al., 2010; Spreng and Grady, 2010; Chen et al., 2013). Hence, while the polarity of effects in the mentalizing system is still under investigation, what is clear is that the same general system mediates the mentalistic processing of the self and others, rather than this being a specific other-based system.

### The Overlap Between Trait Features and Mental States

The mentalizing system has been defined in experiments that require people to think about the mental states of other people and themselves. In many cases, the control condition for such studies is a non-mentalistic “trait judgment” of these same people, such as their gender, physical features, or personality features. However, studies that have looked at trait judgments on their own (rather than as a control condition for mentalizing tasks) or have compared trait judgments directly to mentalizing have yet again shown an overlap in the underlying network. In the context of this overlap, the dmPFC shows some preference for trait judgments compared to mental-state inferences and perhaps even self-trait judgments compared to other-trait judgments (Benoit et al., 2010; Ma et al., 2012; Garrison et al., 2015).

### Self-suppression During Acting

Acting reflects the self-as-other function of the “who” system. As mentioned earlier, the dramaturgical approach to social psychology tells us that, while all people play multiple roles in daily life—for example spouse or employee—these roles are all facets of the self and thus the first-person (1P) perspective. Compared to such everyday role-playing, actors are required to portray other people and to adopt their gestures, emotions, and behaviors. Consequently, actors must think and behave not as themselves but as the characters they are pretending to be on stage. In other words, they have to assume a fictional first-person (Fic1P) perspective. In the only fMRI study of theatrical acting to date, Brown et al. (2019b) sought to identify brain regions preferentially activated when actors adopt a Fic1P perspective during dramatic role-playing. In the scanner, university-trained method actors responded to a series of hypothetical questions from either their own 1P perspective or from that of Romeo (male participants) or Juliet (female participants) from Shakespeare’s drama. Compared to responding as oneself, responding in character as Romeo or Juliet produced global reductions in brain activity. In particular, there were deactivations in the cortical midline network of the frontal lobe, including the dorsomedial and ventromedial prefrontal cortices, areas involved in self-processing, including self-trait processing. Thus, portraying a character through acting seems to be a deactivation-based process, perhaps representing a “loss of self.” It is important to note that the constraints on body movement inherent in neuroimaging experiments precluded the examination of gestural methods of getting into character in Brown et al. (2019b), and so it is not clear if a physical approach to character portrayal would produce the same results.

Another aspect of acting that was not addressed in the Brown et al. (2019b) study is that of self-regulation. The point mentioned earlier that acting involves a split in consciousness between self and character implies that self-regulation is a critical aspect of acting, for example in distinguishing character emotions from self emotions, the latter of which include what Konijn (2000) refers to as “task emotions” about the actor’s task of performing. This taps into executive aspects of self-functioning. Brain areas involved in self-regulation (reviewed in Heatherton, 2011) include not only areas involved in executive control, such as the dorsolateral prefrontal cortex and anterior cingulate cortex, but areas involved in emotion regulation, such as the orbitofrontal cortex, amygdala, and ventromedial prefrontal cortex, the latter of which is involved in mentalizing function as well.

To summarize this section, the “who” system of the human brain contains the well-described mentalizing system at its core. It functions in both production (narration) and perception (mentalizing), making it a mirror system for the cognitive processing of intentionality and intersubjectivity in social communication. The system processes both mental-state and trait information, about both the self and others. This core system receives input from high-level sensory areas in occipito-temporal regions of the brain that process the physical and expressive features of people, as well as input from limbic emotion-appraisal areas that help establish the social and moral status of others in relation to the self, including distinctions such as friend vs. foe and ingroup vs. outgroup.

## Embodied Plot-Schemas: Narrative-Based Cognition

Having described a “who” system for the psychological processing of the self and others as agents in social dramas, I would like to conclude this article by integrating the character-based “who” system with the second major element of narrative theory, namely plot. It has been argued for quite some time that life is similar to the theatre, and vice versa (Goffman, 1959); “all the world’s a stage,” as the saying goes. Storytelling and theatre tap into very fundamental processes of social behavior and social cognition. As mentioned earlier in the article, the protagonists of stories are essentially self-proxies, and stories oftentimes serve as prescriptions for prosocial behavior according to a society’s norms (Scalise Sugiyama, 1996, 2017; Mar and Oatley, 2008; Dunbar, 2014; Wiessner, 2014; Smith et al., 2017; Bietti et al., 2019). Hence, protagonists resemble us and act as we would (or should) in similar situations. In addition Ryan (1980) has argued for a “minimal departure principle” for narrative, which states that storyworlds should tend the resemble the world that we know, rather than some other place that violates our intuitions about social worlds.

Stories are intimately connected with “who” processing, and vice versa. Stories typically display the exploits of people either seeking things that they desire or finding ways to emerge out of chronically oppressive situations to return to a state of normalcy. As a result, the plots of stories generally represent goal-directed behaviors, problem-solving strategies, and coping mechanisms related to obstacles and problems, just as in real life. Problems come in all forms, but literature and drama tend to highlight *social* obstacles first and foremost, i.e., antagonistic interactions and the appraisal of people in our social world as being either friends and foes. From a cultural standpoint, stories tend to be tools for social learning, helping people develop strategies for dealing with real-life problems through the simulations that occur in stories and their plots. The origin of plot-schemas is to be found in people’s natural tendency to seek out the things that they desire and to deal with obstacles that they encounter along the way through problem-solving and conflict-resolution mechanisms. Hence, stories are not just about events or episodes or causal processing *per se*, but about the operations of psychological, protagonist-centered processes related to goals, appraisals, decision making, planning, problem-solving, and so on (Tu and Brown, in press). Perhaps the most artificial aspect of plots is their endpoint-driven nature, since situations in life are far more fluid than they are in stories. Stories always have to end, but events in real life have continuity. Reversals can occur down the road after a period of stasis. The bully who gets beaten up to make the happy ending of an oppression story may come back at a later time to seek revenge on the person who beat him up. People in real life do not live happily ever after.

An important way to integrate “who” processing with plot structure is to conceptualize plot in an intrinsically *character*-based manner as a protagonist-mediated process, something that my colleagues and I have referred to as protagonism (Brown et al., 2019a). Consistent with this, Tu and Brown (in press) developed an Embodied Plot model of literary plot structure, according to which the dramatic arc that is typical of story plots is attributable to psychological mechanisms related to the emotional appraisals of protagonists as they progress through attempts to achieve their goals and employ problem-solving strategies for dealing with obstacles along the way. According to this model, the critical ingredients for creating a plot are not just a series of causally-linked episodes, but also the psychological mechanisms that make these episodes consequential for the protagonist experiencing them. Hence, the sequence of a plot has to be conceived of as a sequence of psychophysiological states experienced by a protagonist, not as a disembodied episodic sequence divorced from human psychology. In particular, the Embodied Plot model argues that the arc-like shape of plot structure is, at least in part, attributable to the arc-like structure of the problem-solving cycle. There is now extensive work in both literary theory and cognitive psychology that argues that stories are first and foremost about the experientiality of people in social scenarios (Fludernik, 1996; Oatley, 1999; Palmer, 2004; Zunshine, 2006; Mar and Oatley, 2008; Hogan, 2011; Herman, 2013).

Having argued that literary plot-structure should be conceived of as plot-schemas that are embodied by a protagonist, I would like to broaden this notion to encompass cognitive psychology more generally and argue that human cognition should be conceived of in the same way as literary plot structure, leading to a concept that I will call narrative-based (NBC) cognition. Hence, I would like to apply the notion of protagonism not just to literary characters but to the self, whereby people during everyday behaviors navigate through life in more or less the same way that a protagonist moves through a story. Much of social behavior, not just in humans but in all animals, is goal-directed behavior that is motivated to satisfy biological and social needs. Goals help formulate action plans, which themselves guide behavior (Schank and Abelson, 1977). Social behaviors are about the strivings and copings of people, just as story plots are. As a result, social behaviors have a story-like structure to them. Everyday life is storied and plotted, just as in literature (Bruner, 1986; Sarbin, 1990). This is not just about autobiographical narratives about one’s past, but about moment-to-moment cognition. I concur with Sarbin (1990) that “the actions of people in daily life are guided by narrative plots, by storylines …” (p. 50).

**Figure 6:**
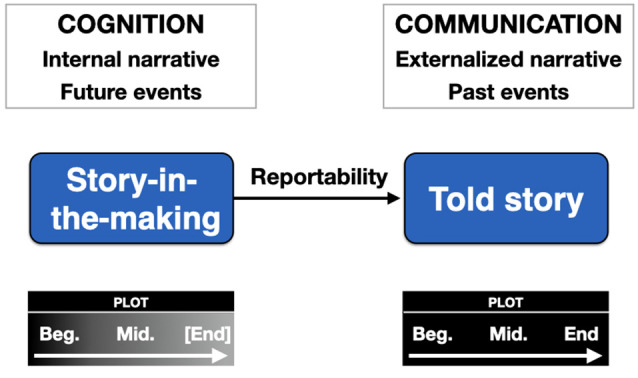
Narrative-based cognition (NBC). The left half of the figure presents a model of cognition based on narrative notions of plot-schemas. This is contrasted with the standard conception of stories in the right half of the figure, where stories are told during acts of communication and deal with past events that are already completed. In the case of cognition, the narratives are internal “stories in the making,” as represented by the incomplete shading inside the plot box. The brackets for [End] suggest that the ending of the story has not yet occurred and can only be simulated by the person through episodic future thinking. Because cognition deals with ongoing narratives, the major focus is on future planning for goal-directed actions, hence prospection. Once such goal-directed plots are completed, they can become reportable as told stories about past events. Abbreviations: Beg, beginning; Mid, middle.

To understand how NBC works, it is critical to place it in comparison to the process of storytelling, as shown in Figure 6. To make the connection to the self more explicit, I will compare NBC to the process of personal narrative, such as when someone tells a personal story about a past event. As shown on the right side of the figure, a personal story is told as a social act of narrative communication to one more audience members, either in person or through media technology. It typically relays information about past events, and is thus based on retrospection. A personal narrative, like a literary narrative, has a standard plot structure to it. In the Aristotelian sense, it has a beginning, middle, and end, where the middle typically presents a complication for the person, one that is resolved in some way by the ending. The story can deal with the self, but it can also deal with other people, including familiar people who are part of one’s social circle, but also strangers, like politicians, celebrities, and other people in the news. Beyond the plot sequence itself, a personal story contains two additional phases that Labov (2001) calls the abstract and the coda. The abstract initiates a bout of storytelling by presenting an overview of the story. It could be something as basic as “You’ll never guess what happened to me at the supermarket today.” At that point, the narrative switches from the present to the past, and the events move into the realm of the storyworld. The storyteller then proceeds to recount the story, typically in a prospective fashion from beginning to middle to end. The coda occurs after the resolution of the complication, and returns the narrative from the past back into the present, and from the storyworld back into the real world of the interlocutors, as in “Can you believe that nonsense? I will never shop there again.”

How does NBC compare to this? If a told story is about something that happened in the past, then NBC is about *a story in the making*, or more accurately a series of overlapping stories in the making at various points of completion. Another way of saying this is that, if a told story is about retrospection, NBC is about prospection, about an emergent story that is unfolding in time in one’s actual life. It is about experiences that are being lived at the present time, compared to the temporal displacement involved in conveying a story about events that took place in the recent or distant past. A told story is an overt behavior that occurs in the context of interpersonal communication, whereas NBC is an *internal* psychological process that occurs as part of the operations of cognition. It is a key component of motivated behavior, whereby desires lead to goals, which lead to action plans, which lead to behaviors aimed at satisfying the desires. Because there are many obstacles to achieving goals, problem-solving strategies have to be activated as part of the process of goal-directed behavior. Decision-making processes require that people weigh the costs and benefits of alternative strategies to achieving the goal. Such decision-making processes require that people simulate the outcomes of these alternative strategies using what psychologists refer to as episodic future thinking (Schacter et al., 2017), by which people visualize not only the future outcome that they desire but also the strategies that they can potentially employ to achieve it. It is telling that the same neural “who” system that is involved in mentalizing about others and the self is also involved in prospective thinking (Buckner and Carroll, 2007; Spreng and Grady, 2010), hence suggesting that the mentalizing system is, at root, a narrative system.

This raises the point that a story-in-the-making, just like a told story, has a beginning, middle, and end, and that it progresses in the same sequence as a told story. However, a defining feature of NBC compared to told stories is that *the ending has not yet occurred*; it can only be imagined using episodic future thinking. NBC truly is an emergent story-in-the-making. Because of this, NBC mainly applies to the self, while a told story can be about the self, other people, or some combination. Whereas told stories are interactive and dialogic, occurring in the form of communicative speech acts, NBC is fundamentally monologic, involving the stream of thought that utilizes inner speech as its primary medium (Wiley, 2016). Because it is internal, NBC lacks the abstract and coda that characterize told stories as speech acts. If a story-in-the-making actually obtains an ending through resolution (whether positive or negative), it can achieve a narrative feature that Labov (2001) refers to as “reportability.” As a result, it can be externalized as a told story about a past event during an act of communication, in most cases as a personal story about the self. As with any personal story, it will convey information about goals, attempts, complications, and resolutions.

While it might seem intuitive that a story-in-the-making progresses temporally from beginning to middle to end, it is not clear why told stories should convey anything more than an ending. It is not clear why told stories should not progress from the end to the middle to the beginning. However, there is no question in my mind that one of the evolutionary design features of our narrative brain is that we convey stories “from the beginning.” As Ryan (2015) pointed out, “when we read a narrative, even one in which the end is presented before the beginning, we adopt the outlook of the characters who are living the plot as their own destiny. Life is lived prospectively and told retrospectively, as Kierkegaard observed, but its narrative replay is once again prospective” (p. 83). This is not simply about preserving chronology to convey the causal structure of the events (e.g., who did what to whom, how it happened), but about the importance of conveying an embodied plot-schema that contextualizes the described events in terms of the intentions, goals, attempts, complications, and consequential outcomes of a protagonist whose welfare and interests are being considered. While the chronology alone might reflect Bruner’s scientific mode of inference based on physical causation, the protagonistic approach to the story reflects Bruner’s narrative mode of inference based on psychological causation. Hence, while a chronological presentation would explain “how” things happened, we need the protagonist’s narrative mode to explain “why” they happened, in other words what motivated the events from a given personal perspective and how their impact was felt. The same series of causally-linked episodes can comprise very different stories for different people based on how the events impact each individual’s personal goals and welfare. As the expression goes, one person’s terrorist is another person’s freedom fighter.

NBC takes full advantage of the mechanisms of social cognition outlined throughout this article. This includes both the standard mode and the narrative mode of social cognition. Given the fact that many, if not most, obstacles to goal achievement are social obstacles, then mechanisms of social cognition are among the most important for engaging in problem-solving processes. At the emotional level, motivational emotions reflect the goal-driven nature of social behavior (Ortony et al., 1988). These can be prospective emotions (e.g., hope, apprehension) or retrospective emotions (e.g., happy, sad). These are protagonist’ emotions that are the driving forces for plots. Hence, we come to understand other people in terms of their NBC and their own embodied plot-schemas, since a critical aspect of the social brain is the realization that other people have minds that are fundamentally similar to our own. Not only are literary characters self-proxies, but so too are the people of our social world. Their stories-in-the-making are understood as being essentially the same as our own, and the process of mentalizing is about inferring how those stories are unfolding in terms of those people’s motivations, goal structures, and agency.

**Figure 7:**
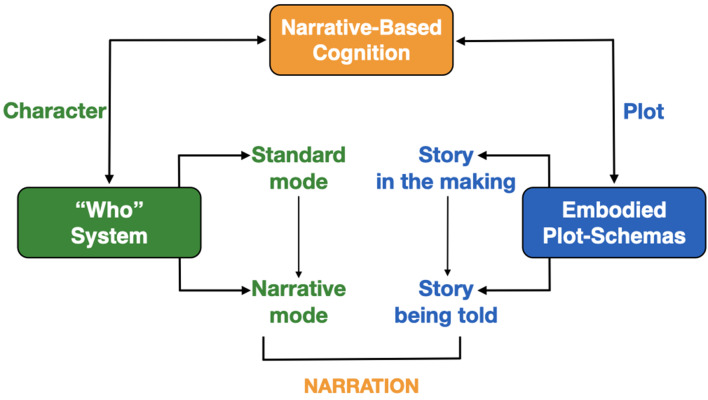
The integration of character and plot in human cognition. The figure integrates information from Figure 4 about the standard and narrative modes of cognition with information from Figure 6 about embodied plot-schemas into a concept of NBC that unifies character and plot in human cognition.

A more complete view of NBC is the scheme shown in Figure 7. NBC employs both a character system (the “who” system”) and a plot system. The character system operates using both the standard mode of social cognition and the narrative mode (both other-as-self and self-as-other). The plot system typically works using stories-in-the-making, but such stories can become reportable when the endpoint for a given goal is reached, where such narratives can be externalized as told stories during acts of communication. This is the process of narration (either first-person or third-person) that was described throughout the article.

## Conclusions

Despite a great deal of work that has been devoted to the study of social cognition and its neural basis, no attempt has been made to characterize a specific “who” system in the brain for the processing of information about the self and others as social agents, one that operates not just for everyday social cognition, but for our experience of the theatrical and literary arts as well. I have attempted to specify such a system, in which the self and others are conceived of as agents in social dramas whose embodied plot-dynamics are driven by a narrative mode of cognition based on mentalistic conceptions of intentions, beliefs, emotions, and desires. The “who” system, in addition to processing the physical and expressive traits of people, processes their mentalistic and narrative features as characters in social dramas. In the standard mode of operation of the “who” system, self-information is processed egocentrically, while other information is processed allocentrically. However, there are also two twists in the operation of this system, resulting in egocentric perception of the other through mentalizing (the other-as-self mechanism) and egocentric production of the other through mimicry (the self-as-other mechanism). The OS system allows us to see other people as proxies of the self and is utilized comparably during production and perception. The OS system is the ultimate mirroring system in human cognition, one that establishes intersubjectivity and parity in communication. The SO system allows us to impersonate others *via* mimicry and acting, capitalizing on the two novel capacities for gestural and vocal imitation that emerged during human evolution, leading to human-specific functions such as pretend to play and dramatic acting. The “who” system’s processing of character information can be combined with embodied plot-schemas about personal narratives to establish NBC as a unifying perspective not just on social cognition but on human cognition more generally.

## Author Contributions

SB conceived the ideas and wrote the manuscript.

## Conflict of Interest

The author declares that the research was conducted in the absence of any commercial or financial relationships that could be construed as a potential conflict of interest.

## Footnotes

^1^I will discuss the perception of other people’s internal mental states and intentionality in the next section about the narrative mode of social cognition.

^2^It is interesting to point out that, in forms of dancing like tango in which there is body contact between the dancers, one’s dance partner becomes a synergistic extension of one’s own body, much the way that hand-held tools are conceptualized in neuroscience (Maravita and Iriki, 2004). Hence, this might be another example of the engulfment of another person in human cognition.
